# Spatially multiplexed single-photon sources based on binary-tree multiplexers with optimized structure

**DOI:** 10.1038/s41598-025-03852-5

**Published:** 2025-05-29

**Authors:** Matyas Mechler, Peter Adam

**Affiliations:** 1 Institute for Solid State Physics and Optics, HUN-REN Wigner Research Centre for Physics, P.O. Box 49, Budapest, 1525 Hungary; 2 Institute of Physics, University of Pécs, Ifjúság útja 6, Pécs, 7624 Hungary

**Keywords:** Single photons and quantum effects, Quantum information

## Abstract

We develop a method for optimizing the structure of general binary-tree multiplexers realized with asymmetric photon routers aiming at improving the performance of spatially multiplexed single-photon sources. Our procedure systematically considers all possible binary-tree multiplexers that can be constructed using a certain number of photon routers. Using this method one can select the multiplexer structure that leads to the highest single-photon probability for a given set of loss parameters characterizing the system. We determine the optimal general binary-tree multiplexers for experimentally realizable values of the transmission coefficients of the photon routers and that of the detector efficiency. We show that single-photon sources based on such optimal multiplexers yield higher single-photon probabilities than what can be achieved with single-photon sources based on any other spatial multiplexer considered in the literature. Our approach improves the performance of multiplexed single-photon sources even for small system sizes which is the typical situation in current experiments.

## Introduction

The substantial role of single-photon sources (SPSs) in the effective realization of a number of experiments in the fields of quantum information processing and photonic quantum technology keeps their development in the focus of research^1–3^. Multiplexed SPSs can be promising candidates for yielding indistinguishable single photons in near-perfect spatial modes with known polarization on demand. Such sources are based on heralded SPSs^4–10^ in which the detection of one member of a correlated photon pair generated in nonlinear optical processes heralds the presence of its twin photon. In heralded SPSs, the multiphoton noise originating from the inherent probabilistic nature of the nonlinear processes can be reduced by using single-photon detectors with photon number resolving capabilities for heralding, or by decreasing the mean photon number of the generated photon pairs. Multiplexing several sources of heralded photons can compensate for the decrease of the probability of successful heralding caused by the reduction of the mean photon number. Multiplexing can be realized by suitable switching devices in which heralded photons generated in particular multiplexed units are rerouted to a single output mode. Various schemes have been proposed for SPSs based on spatial^11–22^ and temporal multiplexing^18,23–34^, and some of them have been successfully implemented in experiments^13,14,16,17,20,21,27,29,30,32,35^.

In real multiplexed SPSs, the presence of various losses leads to the degradation of the performance^15,19^. The output single-photon probability of these systems can be maximized by determining the optimal number of multiplexed units and the mean number of photon pairs generated in the units. The optimization can be performed by applying the full statistical theories developed for the description of such systems^18,22,36,37^. According to the analyses, state-of-the-art multiplexed SPSs realized with low-loss optical elements can yield high single-photon probabilities with low multiphoton contribution^22,36–38^.

In spatially multiplexed SPSs, several individual pulsed heralded photon sources are applied in parallel. These sources can be realized by using physically separate nonlinear processes or in separate spatial modes of a single process. After a successful heralding event in one of the heralded sources, a spatial multiplexer composed of a set of binary photon routers is used to reroute the corresponding heralded signal photons to a single output. Special types of spatial multiplexers considered thus far in the literature are symmetric (complete binary-tree)^13,14,17,20,21^, asymmetric (chain-like)^15,19,37^, and incomplete binary-tree multiplexers^22,38^. Successful experimental realizations of multiplexed SPSs based on symmetric multiplexers have been reported up to four multiplexed units by using spontaneous parametric down-conversion in bulk crystals^13,21^ and waveguides^17^, and by using spontaneous four-wave mixing up to two multiplexed units in photonic crystal fibers^14,16,20^. Theoretical analyses showed that a particular multiplexer structure can outperform the other for a certain range of the loss parameters when applied in SPSs^22,38^. Hence, finding novel multiplexing schemes that can further improve the performance of SPSs is an important goal of the researches on multiplexed SPSs.

In the present paper, we consider SPSs based on general binary-tree multiplexers. Accordingly, we treat all possible binary-tree multiplexers that can be constructed using a given number of binary photon routers. We develop a systematic method for finding the optimal binary-tree structure that leads to a SPS with the highest performance for a given set of loss parameters. We analyze the performance of SPSs based on general binary-tree multiplexers with optimal structure in detail. We show that single-photon sources based on optimal general binary-tree multiplexers yield higher single-photon probabilities than that can be achieved with single-photon sources based on any special spatial multiplexer considered in the literature thus far.

## Single-photon sources based on general binary-tree multiplexers

A SPS based on a general spatial multiplexer contains a set of multiplexed units (MUs) and a multiport routing device called multiplexer. The MUs are heralded SPSs independent of each other. Each MU contains a nonlinear photon pair source and a detector for detecting the idler photons of the photon pairs. Detection events in the MUs herald the presence of the corresponding signal photons which in turn are directed to a single output by the multiplexer. In this paper, we consider general spatial multiplexers built of binary photon routers (PRs) which are routing elements with two inputs and a single output. In spatially multiplexed single-photon source experiments, several types of optical switching devices can be used as photon routers. The most known types are bulk electro-optic polarization rotating switches^13,21,22^, integrated opto-ceramic switches^14,16^, and electro-optic switches^17,35^. PRs are generally asymmetric: the photon losses characterizing the two input ports of the PR differ. In ref.^22^, the terms *transmission* and *reflection efficiencies* were used for the efficiencies characterizing the two input ports of the PR with the corresponding notations $V_t$ and $V_r$, respectively. In the present paper, we keep these notations for the inputs of the PRs and use the term *transmission coefficients* to refer to both of them.

The output of a PR can be connected to any of the inputs of another PR. Several building logics for realizing spatial multiplexers consisting of binary photon routers have hitherto been analyzed in the literature resulting in different types of multiplexers, such as symmetric multiplexers also referred to as complete binary-tree multiplexers, asymmetric (chain-like) structures, and various incomplete binary-tree multiplexers constructed by following either a geometric logic or a transmission-based logic. Now we do not pose any restrictions on the structure of the multiplexer, we consider all possible binary trees that can be constructed by using a certain number of PRs. Consequently, we introduce the term *general binary-tree multiplexer* (GBM) to refer to these multiplexers. We note that periodicity of the single-photon output is a requirement posed by most applications that can be ensured by pulsed pumping of the source generating the photon pairs. Also, beside multiplexing, suppressing multiphoton noise in multiplexed SPSs can be guaranteed by applying single-photon detectors with photon-number-resolving capabilities in the MUs^39–45^, therefore we assume such detectors in our calculations. We note that the repetition frequency of spatially multiplexed SPSs is limited by the deadtime of the detectors. A method based on a multiplexed detector array has already been developed for reducing deadtime^46,47^. We also mention that the MUs can contain an optional delay line placed into the path of the signal photon that is responsible for introducing a sufficiently long delay into the traveling time of the photon before it enters the multiplexer. This delay enables the operation of the logic controlling the routers.

**Fig. 1:**
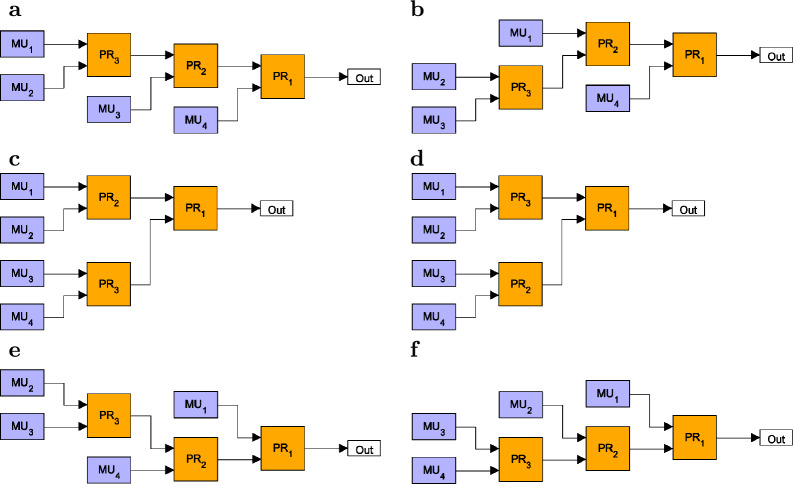
All binary-tree multiplexers constructed by using three binary PRs. The corresponding integer sequences identifying the particular multiplexers are: (**a**) [1,1,1], (**b**) [1,1,2], (**c**) [1,1,3], (**d**) [1,2,1], (**e**) [1,2,2], (**f**) [1,2,3].

Our aim is to find the GBM having *N* inputs and, consequently, composed of $N-1$ identical asymmetric PRs that gives the highest output single-photon probability $P_1$ when applied in a multiplexed SPS. Therefore, we need to test all possible different binary-tree structures. We assume that the positioning of the PRs in a binary-tree is fixed, that is, the inputs of all routers characterized by given transmission efficiencies are in the same geometric position in the tree. We prescribe that the numbering of the inputs of the multiplexer in any stage of the construction follows the geometric rule applied at the inputs of the first router, e.g., from top to bottom in the figure of the multiplexer. Then we can represent a binary-tree multiplexer comprising $N-1$ PRs by a sequence of integer numbers of length $N-1$ according to the following logic. The first number is always 1 showing that the first router is connected to the single output of the multiplexer. The second number identifies the connection point of the second router to the first one, therefore it can take the values 1 or 2. The *n*th number is the connection point of the *n*th PR to any of the input ports of the multiplexer created in the previous steps. As this multiplexer was built of $n-1$ PRs, hence it has *n* inputs and, accordingly, the *n*th number in the sequence of integer numbers can take any integer value between 1 and *n*. Obviously, the number of such sequences for binary-tree multiplexers having *N* inputs and formed by $N-1$ PRs is $(N-1)!$. As an example, in Fig. 1 we show all the possible multiplexers comprising three PRs, and in the caption of the Figure we specify the integer sequences identifying the various multiplexers. These sequences representing the six multiplexers in the Figure are [1,1,1], [1,1,2], [1,1,3], [1,2,1], [1,2,2], [1,2,3]. However, as we assume identical routers, the sequences [1,1,3] and [1,2,1] represent the same two-level complete binary-tree multiplexers. To avoid this problem, we apply the following rule: we accept only sequences containing 0 or 1 increment or arbitrary decrement between subsequent elements. This way, the sequence [1,1,3] is excluded from the above list of sequences. It can be shown that, following this rule, those sequences representing binary-tree multiplexers having structures identical with an already observed one can be excluded from the list of sequences for any number of PRs. The number of sequences of length $N-1$ generated in this way provides the number $K_N$ of different binary-tree multiplexers having *N* inputs. It can be found that $K_N$ can be calculated as

1 $$\begin{aligned} K_{N}=\prod _{k=2}^{N-1}\frac{N+k-1}{k} \quad \text {for }N\ge 3. \end{aligned}$$

We note that the number $K_{N+1}$ is known as the *N*th Catalan number^48^.

As it was explained above, a binary PR is a two-port routing device that can be characterized by the transmission coefficients $V_t$ and $V_r$. Similarly, a multiplexer which is a multiport routing device can be characterized by the *total transmission coefficients* $V_n$ describing the transmission probabilities between each input and the output of the multiplexer. These total transmission coefficients are in fact products of the transmission coefficients of the PRs, hence they can be written in the symbolic form of

2 $$\begin{aligned} V_n=V_bV_r^jV_t^k\qquad (0\le j,k\le N). \end{aligned}$$

Here, the multiplicative factor $V_b$ termed as *general transmission coefficient* characterizes all other losses experienced by the photons while propagating to the input of the multiplexer after their heralding. A multiplexer having *N* inputs can be characterized by *N* total transmission coefficients. The number *N* is equal to the number of multiplexed units that are connected to the given multiplexer. In the next section, we will compare our results to the performance of SPSs based on asymmetric (ASYM) multiplexers. Such multiplexers have a chain-like structure characterized by the total transmission coefficients

3 $$\begin{array}{*{20}l} {V_{n} = V_{b} V_{1} V_{2}^{{n - 1}} } & {{\text{if}}\quad n < N,} \\ {V_{n} = V_{b} V_{2}^{{n - 1}} } & {{\text{if}}\quad n = N,} \\ \end{array}$$

where $V_1$ and $V_2$ are the smaller and larger, respectively, of the transmission coefficients $V_r$ and $V_t$.

For analyzing SPSs based on GBMs, we will apply the general statistical theory developed previously for treating SPSs based on either spatial or temporal multiplexing equipped with photon-number-resolving detectors realizing any detection strategy^22,36^. We will consider only ranges of the loss parameters for which single-photon detection is certainly the optimal detection strategy. In this case, the probability $P_i$ of obtaining *i* photons at the output of multiplexed SPSs can be written as

4 $$\begin{aligned} P_i=\left( 1-P^{(D)}_1\right) ^N\delta _{i,0}+\sum _{n=1}^N\left[ \left( 1-P^{(D)}_1\right) ^{n-1}\times \sum _{l=i}^\infty P^{(D)}(1|l)P^{(\lambda )}(l)V_n(i|l)\right]. \end{aligned}$$

Here, the variable *l* denotes the number of photon pairs generated by the nonlinear source in the *n*th multiplexed unit $\hbox {MU}_n$, and *N* is the number of multiplexed units in the SPS. $P^{(\lambda )}(l)$ is the probability of generating *l* pairs in a MU assuming that the mean photon number of the generated pairs, that is, the *input mean photon number* is $\lambda$. We assume that a single-mode nonlinear process with strong spectral filtering is used in the scheme. In this case, the multiplexed SPSs can yield highly indistinguishable single photons that are required in many experiments and applications^14,21,49^, and the probability distribution of the input mean photon number is thermal:

5 $$\begin{aligned} P^{(\lambda )}(l)=\frac{\lambda ^l}{(1+\lambda )^{1+l}}. \end{aligned}$$

$P^{(D)}(1|l)$ denotes the conditional probability of registering a single photon provided that *l* photons arrive at the detector with detector efficiency $V_D$. It can be expressed as

6 $$\begin{aligned} P^{(D)}(1|l)=lV_D(1-V_D)^{l-1}. \end{aligned}$$

The total probability $P^{(D)}_1$ of the event that a single photon is detected can be derived as

7 $$\begin{aligned} P^{(D)}_1=\sum _{l=1}^\infty P^{(D)}(1|l)P^{(\lambda )}(l)=\frac{V_D\lambda }{(V_D\lambda +1)^2}. \end{aligned}$$

In our calculations, the probabilities $P^{(D)}_1$, $P^{(D)}(1|l)$, $P^{(\lambda )}(l)$, and the input mean photon number $\lambda$ are assumed to be independent of the sequential number *n* of the MU.

Finally, $V_n(i|l)$ is the conditional probability of the event that *i* photons reach the output of the multiplexer provided that *l* signal photons arrive from the *n*th multiplexed unit $\hbox {MU}_n$ into the system. This probability is expressed as

8 $$\begin{aligned} V_n(i|l)=\left( {\begin{array}{c}l\\ i\end{array}}\right) V_n^i(1-V_n)^{l-i}, \end{aligned}$$

where the total transmission coefficient $V_n$ characterizes the losses of the *n*th arm of the particular multiplexer.

From the second term of Eq. (4), one can see that this theory assumes a priority logic controlling the multiplexed SPS that prefers the MU with the smallest sequential number *n* if multiple heralding events happen in different MUs. It seems plausible that by assigning smaller sequential numbers *n* to arms with higher total transmission coefficients $V_n$, the achievable single-photon probability $P_1$ can be higher. Consequently, it is reasonable to choose a numbering for the MUs for which the associated total transmission coefficients $V_n$ are arranged into a decreasing order, that is, $V_1\ge V_2\ge \dots \ge V_N$. Obviously, the numbering of the multiplexer arms having identical total transmission coefficients is arbitrary.

Knowing the probabilities $P_i$ from Eq. (4), the normalized second-order autocorrelation function can be obtained as

9 $$\begin{aligned} g^{(2)}(t=0)=\frac{\displaystyle \sum\nolimits _{i=2}^\infty P_i i (i-1)}{\left( \displaystyle \sum\nolimits _{i=1}^{\infty } P_i i\right) ^2}. \end{aligned}$$

This function quantifies the contribution of multiphoton components in the output state compared to that of the single-photon component. In the next section, we also present results on this quantity. Note that spatially multiplexed single-photon sources realized in experiments are generally tested for their single-photon probability and for the multiphoton components of the output signal characterized by the normalized second order autocorrelation function^2^. As it was outlined in experimental papers on the topic, the single-photon probability can be measured by photon-number-resolving detectors capable of detecting single photons, while the normalized second order autocorrelation function can be measured by a standard Hanbury–Brown–Twiss setup^13,14,27,50,51^.

**Fig. 2:**
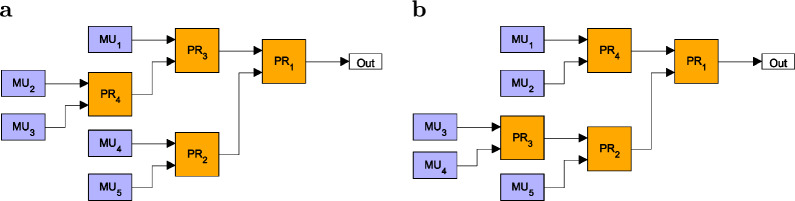
Two binary-tree multiplexers constructed by using four binary PRs having the same sets $\{V_n\}$ of total transmission coefficients $V_n$. The corresponding integer sequences identifying the particular multiplexers are: (**a**) [1,2,1,2], (**b**) [1,2,2,1]. Assuming that the transmission coefficients of the upper and lower inputs of the PRs are denoted by $V_t$ and $V_r$, respectively, the corresponding sets are (**a**) $\{V_t^2,V_t^2V_r,V_tV_r^2,V_tV_r,V_r^2\}$ and (**b**) $\{V_t^2,V_tV_r,V_t^2V_r,V_tV_r^2,V_r^2\}$.

The described statistical theory can be used for the optimization of multiplexed SPSs aiming at determining the optimal number of multiplexed units $N_{\text {opt}}$ and the optimal input mean photon number $\lambda _{\text {opt}}$ corresponding to the maximal value of the output single-photon probability $P_{1,\max }$. The optimum exists because the function $P_1(N,\lambda )$ describing the single-photon probability against the number of multiplexed units and the input mean photon number has a global maximum for most of the systems. The common characteristics of such systems is that the transmission efficiencies of the various arms change, generally decrease, by increasing the number of PRs in the system. Typical examples are the symmetric and the incomplete multiplexers. In contrast, for asymmetric (chain-like) multiplexers the same function $P_1(N,\lambda )$ monotonically increases with the number of multiplexed units and it eventually saturates^15,19,37^. In such systems $N_\text {opt}$ can be chosen so that the corresponding value of $P_1(N_\text {opt},\lambda _\text {opt})$ is reasonably close to the saturated value.

The task for SPSs based on GBMs is to determine the optimal structure for a given number of multiplexed units *N*. In this paper we determine the optimal structures for a predefined number of multiplexed units *N*. Hence, we do not address the problem of finding an optimal number of multiplexed units $N_\text {opt}$ for SPSs based on GBM.

Finding the optimal structure for a given number *N* can be realized as follows. First, we generate the sequences representing all GBM structures comprising $N-1$ routers by applying a specific systematic rule. Based on these sequences, it is possible to calculate the corresponding sets $\{V_n\}$ of total transmission coefficients $V_n$ characterizing the particular multiplexers. At this point, we mention that some of the sets can contain the same symbolic total transmission coefficients for geometries characterized by different sequences. As an example, Fig. 2 shows two different geometries for $N=5$ for which the sets $\{V_n\}$ are identical. In this case, by assuming that the transmission coefficients of the upper and lower inputs of the PRs are denoted by $V_t$ and $V_r$, respectively, the corresponding sets are a) $\{V_t^2,V_t^2V_r,V_tV_r^2,V_tV_r,V_r^2\}$ and b) $\{V_t^2,V_tV_r,V_t^2V_r,V_tV_r^2,V_r^2\}$. Recall that the list of $V_n$s are sorted to a decreasing order before the optimization, therefore SPSs based on multiplexers with identical sets $\{V_n\}$ exhibit the same performance. For this reason, we consider only the GBM structure appearing as the first one in our logic in the case of identical $\{V_n\}$s. As a consequence, the number of multiplexers with physically different structures is lower than the number $K_N$ defined in Eq. (1) in the case of multiplexers having *N* inputs.

After determining the set of total transmission coefficients, we can apply Eq. (4) to maximize the single-photon probability $P_{1,S}(\lambda )$ of the SPS based on the given GBM, where we use the subscript *S* in $P_1$ for denoting the structure. As in our case the number *N* of MUs is fixed, the input mean photon number $\lambda$ is the only variable that can be optimized for given values of the transmission coefficients $V_r$ and $V_t$ and the detector efficiency $V_D$. As the function $P_{1,S}(\lambda )$ has a single maximum, any method for finding extremums can be used to determine the optimal value of $\lambda$. Finally, after determining the single-photon probability $P_{1,S}(\lambda _\text {opt})$ that can be achieved for particular $\lambda _\text {opt}$ values for all possible structures *S*, we find the highest one denoted by $P_{1,\max }$. The structure *S* corresponding to this maximal achievable single-photon probability $P_{1,\max }$ is said to be the optimal structure $S_\text {opt}$ of the multiplexer for the number of multiplexed units *N*. The GBM with optimal structure will be termed *optimal general binary-tree multiplexer* and abbreviated as OGBM. Using this method, one can determine the OGBM for any set of loss parameters characterizing the SPS.

The proposed method can be applied for any number of multiplexed units *N* to determine the optimal multiplexing structure. Hence, it can be used to design arbitrary SPS experiments based on OGBMs. From the point of view of applicability, a relevant question is how the method scales with increasing numbers of multiplexed units. Basically, the computational requirements of the method scale with the number of different binary tree structures determined by the numbers $K_N$ defined in Eq. (1). These numbers can be considerably high for higher values of the number of multiplexed units *N*, for example, for $N=11$ and $N=16$ they are $K_{N=11}=16796$ and $K_{N=16}\approx 9.69\times 10^6$, respectively. However, as we have pointed out above, the actual computational requirements of the method scale with the number of different sets of the total transmission coefficients $\{V_n\}$. We have determined the numbers of different sets of $\{V_n\}$ for the previous examples. We have found that the numbers of physically different structures for $N=11$ and $N=16$ are only 7624 and $\approx 1.93\times 10^6$. It means that only 45% and 20% of the amount of calculations predicted by the corresponding number $K_N$ is sufficient to optimize the structure of a multiplexer formed by 10 and 15 photon routers, respectively. The reduction for higher number of photon routers is probably even higher. Albeit the application of the proposed method can be cumbersome for higher values of the number of multiplexed units *N*, it can still be used to determine the optimal multiplexer structure for any number *N*, its applicability solely depends on the computational capacity of the available computers. In a recent paper^3^ it was found that for SPSs based on previously studied spatial multiplexers, the optimal values of the numbers of multiplexed units are relatively low, $N_{\text {opt}}=20,\dots ,30$, for loss parameters that can possibly be realized in current experiments. Note that for higher losses the value of the optimal number of multiplexed units $N_{\text {opt}}$ decreases. Hence, we believe that for specific sets of the loss parameters even the full optimization can be realized with well-designed codes and computers with sufficiently high computational power. We note that the computational task may be reduced by the application of certain machine learning methods. This possibility deserves consideration in the future.

## Results

In this section, we present our results on the optimization of SPSs based on GBMs composed of general asymmetric routers. Our goal for SPSs based on GBMs is to find the optimal structure for a given number of multiplexed units *N* that has the best performance. Therefore we confine our calculations to high transmission and detector efficiencies that can be realized experimentally with state-of-the-art devices. Hence, in this section the detector efficiency is set to $V_D=0.95$, the highest value reported in ref.^41^. However, we apply different values $V_D$ in certain cases we consider as relevant. The general transmission coefficient is set to $V_b=0.98$ in all our calculations, hence generally we do not indicate this value in the following. Routers built of bulk-optical elements exhibited the highest transmission efficiencies $V_r=0.99$ and $V_t=0.985$ reported in refs.^32,52^ These values are applied in our analysis whenever individual parameter sets or sweeps for other parameters are analyzed. However, we use $V_r=V_t=0.99$ as the upper boundaries of the ranges of these efficiencies whenever we present parameter sweeps for them to show the symmetry of these parameters, while the lower boundaries of these ranges are chosen to be $V_r=V_t=0.9$ ensuring that single-photon detection yields the highest single-photon probability for the whole considered parameter range (see, e.g., ref.^37^). As already mentioned in the Introduction, spatially multiplexed SPSs have been realized up to four multiplexed units. Earlier theoretical analyses have shown that high single-photon probabilities can be achieved with spatially multiplexed SPSs even with suboptimal system sizes. Hence, analyzing such systems is physically relevant. In the present paper, we consider spatially multiplexed SPSs with $N=11$ multiplexed units as an example for the application of the proposed method.

**Fig. 3:**
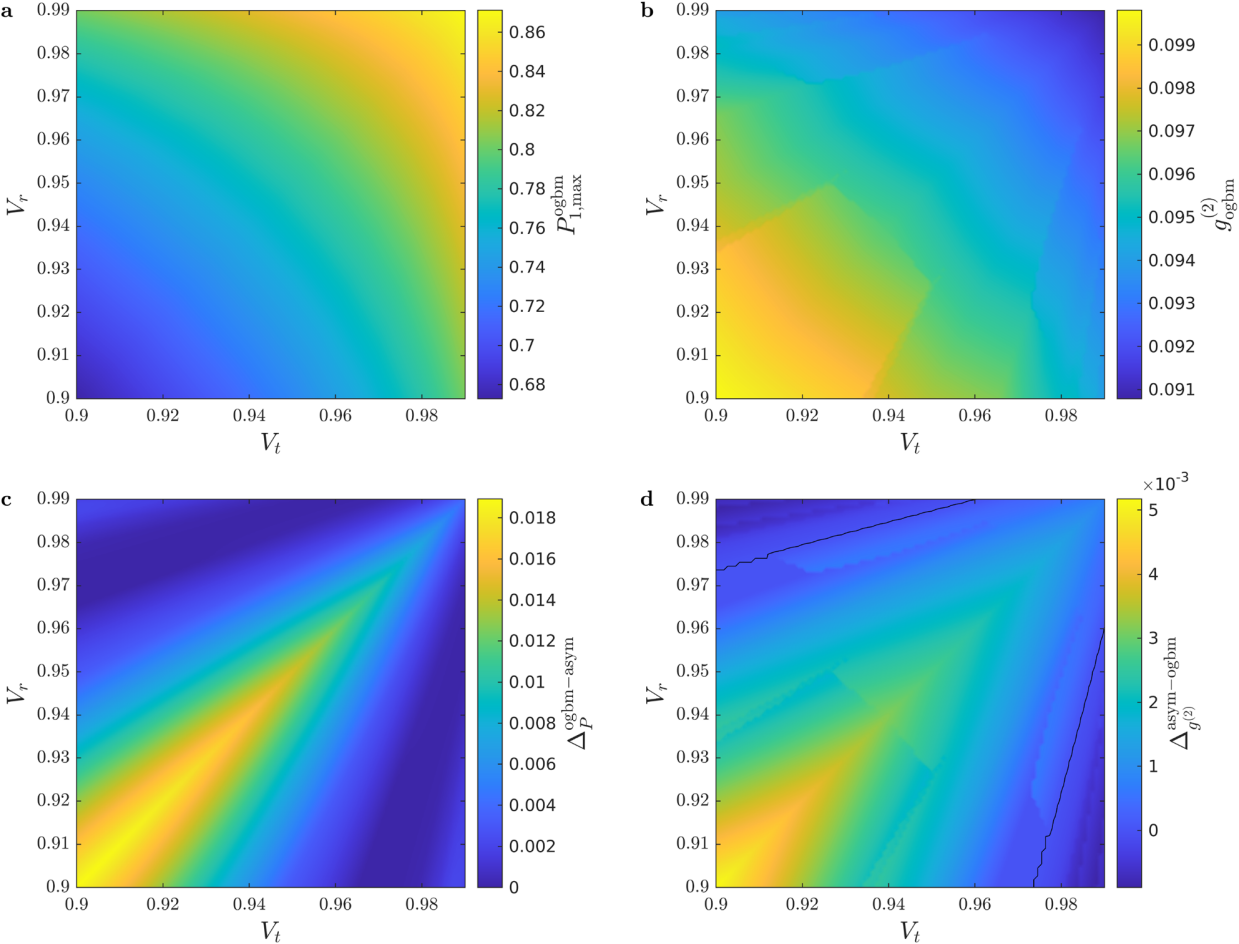
(**a**) The maximal single-photon probability $P_{1,\max }^{\textrm{ogbm}}$ and (**b**) the normalized second-order autocorrelation function $g^{(2)}_{\textrm{ogbm}}$ for SPSs based on OGBMs as functions of the transmission coefficients $V_t$ and $V_r$. (**c**) The difference $\Delta _P^{\mathrm{ogbm - asym}}$ between the maximal single-photon probabilities, and (**d**) the difference $\Delta _{g^{(2)}}^{\mathrm{asym - ogbm}}$ between the normalized second-order autocorrelation functions for SPSs based on OGBM and ASYM multiplexers, respectively, as functions of the transmission coefficients $V_t$ and $V_r$. Here the detector efficiency $V_D=0.95$ and the number of multiplexed units $N=11$.

Figure 3a, b present the maximal single-photon probability $P_{1,\max }^{\textrm{ogbm}}$ and the corresponding normalized second-order autocorrelation function $g^{(2)}_{\textrm{ogbm}}$, respectively, for SPSs based on OGBMs, while Fig. 3c, d show the difference $\Delta _P^{\mathrm{ogbm - asym}}$ between the maximal single-photon probabilities and the difference $\Delta _{g^{(2)}}^{\mathrm{asym - ogbm}}$ between the normalized second-order autocorrelation functions, respectively, for SPSs based on OGBM and ASYM multiplexers as functions of the transmission coefficients $V_t$ and $V_r$ for the detector efficiency $V_D=0.95$, and the number of multiplexed units $N=11$. It can be seen that, as it is expected, the maximal single-photon probability $P_{1,\max }$ is higher while the second-order autocorrelation function $g^{(2)}$ is better, that is, lower for higher values of the transmission coefficients of the PRs. The highest maximal single-photon probabilities in this region are above $P_{1,\max }>0.86$ and the lowest corresponding values of the second-order autocorrelation function are $g^{(2)}<0.1$. Figure 3c shows that using SPSs based on OGBMs give higher single-photon probabilities than SPSs based on ASYM multiplexers for the whole considered parameter range. From Fig. 3d one can deduce that the normalized second order autocorrelation function values $g^{(2)}$ are smaller for SPSs based on OGBMs than for SPSs based on ASYM multiplexers except for very asymmetric PRs, that is, for $V_r\gg V_t$ or $V_r\ll V_t$. The $g^{(2)}$ values can be lower for SPSs based on ASYM multiplexers than for those based on OGBMs because the optimization was carried out for the single-photon probability $P_1$. We have also compared our results with output-extended incomplete binary-tree multiplexers and symmetric multiplexers for $N=4$ and $N=8$, and we have found that the advantage of SPSs based on OGBMs is on the same range as in the case of ASYM multiplexers.

**Fig. 4:**
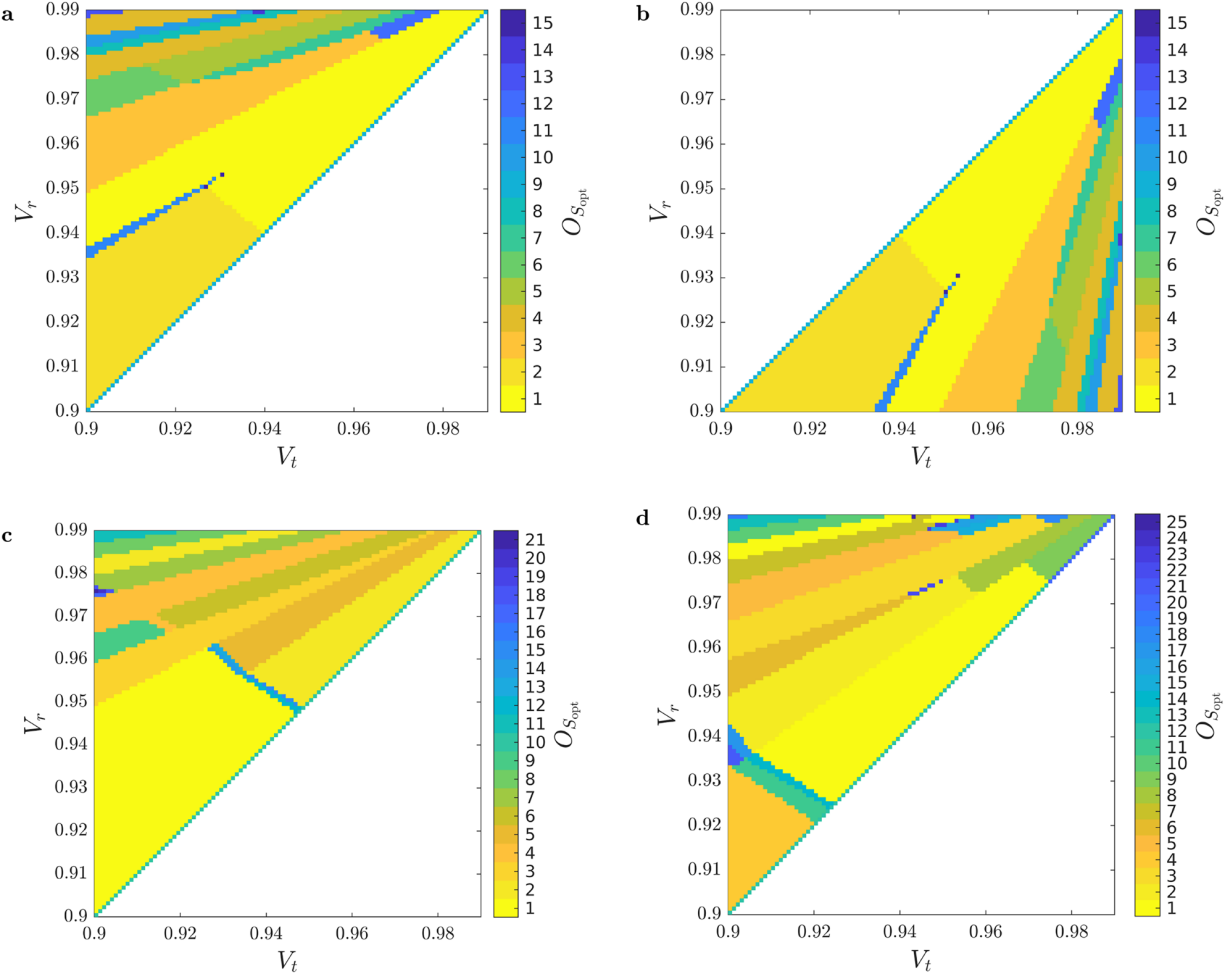
Occurrence $O_{S_\text {opt}}$ of the various optimal multiplexer structures $S_\text {opt}$ for SPSs based on OGBMs for (**a**) the detector efficiency $V_D=0.95$ and $V_r\ge V_t$, (**b**) $V_D=0.95$ and $V_r\le V_t$, (**c**) $V_D=0.85$ and $V_r\ge V_t$, and (**d**) $V_D=0.8$ and $V_r\ge V_t$, for the number of multiplexed units $N=11$. A particular color denotes a given structure. Increasing sequential numbers of $O_{S_\text {opt}}$ in the color bar represent decreasing occurrence of a specific structure.

Next, we show our results on the optimal multiplexer structures yielding the maximal single-photon probabilities. Figure 4 presents *occurrence* of the various optimal multiplexer structures $S_\text {opt}$: an ordinal number $O_{S_\text {opt}}$ is assigned to various structures with an ordering described later. This is plotted for SPSs based on OGBMs for the detector efficiency $V_D=0.95$ and $V_r\ge V_t$ (Fig. 4a), $V_D=0.95$ and $V_r\le V_t$ (Fig. 4b), $V_D=0.85$ and $V_r\ge V_t$ (Fig. 4c), and $V_D=0.8$ and $V_r\ge V_t$ (Fig. 4d), for the number of multiplexed units $N=11$. The meaning of the occurrence is the following. Each color in a subfigure of Fig. 4 corresponds to a specific multiplexer structure. Thus, a region in the $V_t-V_r$ plane displayed with a given color indicates that the corresponding multiplexer structure is optimal for all the loss parameter pairs in the region. The numbers at the color bars in Fig. 4 are sequential numbers $O_{S_\text {opt}}$ of the occurrences corresponding to the decreasing order of the sizes of the areas occupied by the various colors in the $V_r-V_t$ plane. Accordingly, the sequential number 1 assigned to the color yellow corresponds to the largest area and colors indicated by higher numbers cover smaller areas in the figure. Hence, the multiplexer structure denoted by yellow is the most frequent in the $V_r-V_t$ plane. Colors with increasing sequential numbers $O_{S_\text {opt}}$ represent decreasing occurrence of the corresponding structures. Note that any color can denote different structures for the different subfigures as the value of the detector efficiency $V_D$ is different for each subfigure that can lead to different optimal structures.

**Fig. 5:**
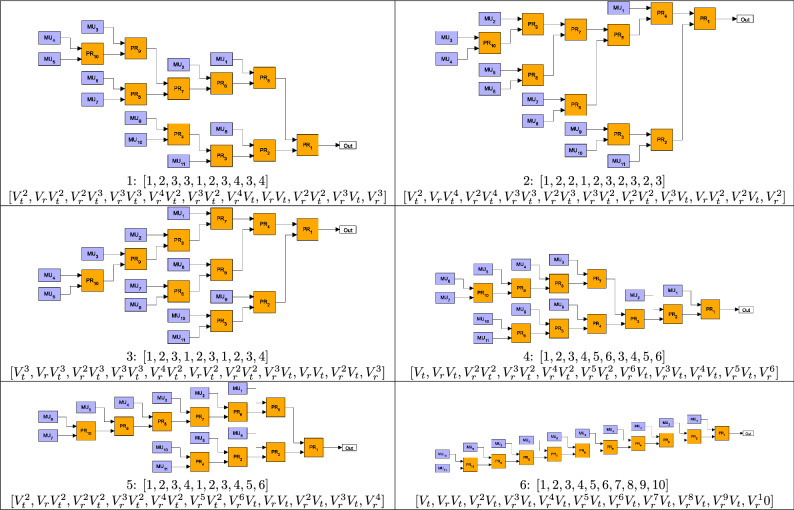
The six most frequent optimal structures of OGBMs in the region $V_r\ge V_t$ for the detector efficiency $V_D=0.95$ and the number of multiplexed units $N=11$. $V_t$ and $V_r$ correspond to the upper and lower inputs, respectively, of the individual PRs. The sequential numbers of the occurrence $O_{S_\text {opt}}$ followed by the integer sequences representing the structures, and the lists of the total transmission coefficients $V_n$ in the order of the multiplexed units $\hbox {MU}_n$ are presented below the structures.

**Fig. 6:**
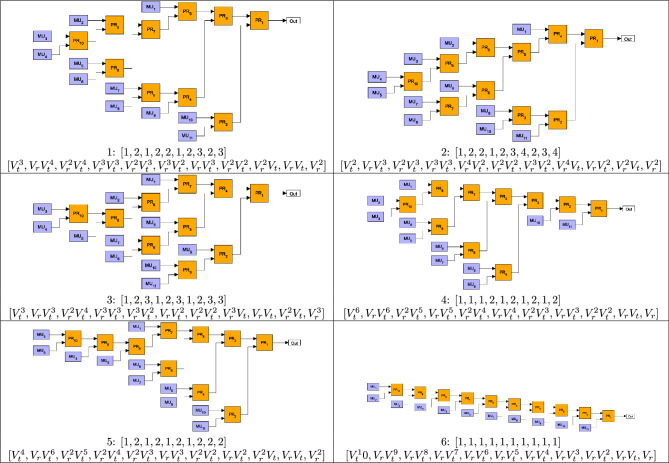
The six most frequent optimal structures of OGBMs in the region $V_r\le V_t$ for the detector efficiency $V_D=0.95$ and the number of multiplexed units $N=11$. $V_t$ and $V_r$ correspond to the upper and lower inputs, respectively, of the individual PRs. The sequential numbers of the occurrence $O_{S_\text {opt}}$ followed by the integer sequences representing the structures, and the lists of the total transmission coefficients $V_n$ in the order of the multiplexed units $\hbox {MU}_n$ are presented below the structures.

The sizes and shapes of the regions in Fig. 4a, b are identical but mirrored to the $V_t=V_r$ line (they are reflected congruent shapes) as it is expected from symmetry consideration. However, the identified structures belonging to a particular color in the two regions can be different due to the fact that the method described in section “Single-photon sources based on general binary-tree multiplexers” selects a single GBM structure out of those having identical sets $\{V_n\}$ of total transmission coefficients. This can be deduced from Figs. 5 and 6 where the six most frequent optimal structures of OGBMs occurring in Fig. 4a (region $V_r\le V_t$) and 4b (region $V_r\ge V_t$) are presented, respectively, for the detector efficiency $V_D = 0.95$ and the number of multiplexed units $N = 11$. In these figures the transmission coefficients $V_t$ and $V_r$ correspond to the upper and lower inputs, respectively, of the individual PRs. The figures also contain the sequential numbers of the occurrence $O_{S_\text {opt}}$ of the structures shown in Fig. 4a, b, the integer sequences representing the structure, and the lists of the total transmission coefficients $V_i$ in the order of the multiplexed units $\hbox {MU}_n$. Note that the sets of total transmission coefficients $\{V_n\}$ in Fig. 5 are the same as the corresponding sets in Fig. 6 if the roles of the transmission coefficients $V_r$ and $V_t$ are swapped. This property reflects the expected symmetry mentioned before. Figures 5 and 6 show that the asymmetric multiplexer having a chain-like structure proves to be the best for a certain region of the transmission coefficients $V_t$ and $V_r$. Obviously, in this region the difference $\Delta _P^{\mathrm{ogbm - asym}}$ presented in Fig. 3c is zero.

**Fig. 7:**
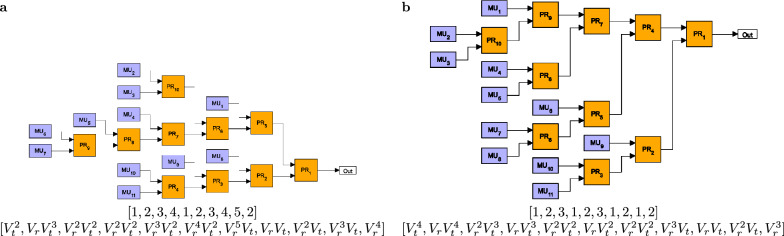
Optimal structures of OGBMs for the detector efficiencies (**a**) $V_D=0.8$ and (**b**) $V_D=0.85$, the transmission coefficients $V_r=0.99$ and $V_t=0.985$, and the number of multiplexed units $N=11$. The integer sequences representing the structures and the lists of the total transmission coefficients $V_n$ in the order of the multiplexed units $\hbox {MU}_n$ are presented below the structures.

From Fig. 4c, d one can deduce that by decreasing the value of the detector efficiency $V_D$ the number of optimal multiplexer structures occurring in the analyzed domain of the parameters $V_r$ and $V_t$ increases, and the regions representing particular structures are considerably different. Obviously, the identified optimal structures can be different for different values of the detector efficiency $V_D$ even for given values of the transmission coefficients $V_r$ and $V_t$. As an example, in Fig. 7 we show the optimal structures of OGBMs for the detector efficiencies $V_D=0.8$ (Fig. 7a) and $V_D=0.85$ (Fig. 7b), for the transmission coefficients $V_r=0.99$ and $V_t=0.985$, and for the number of multiplexed units $N=11$. The integer sequences representing the structures and the lists of the total transmission coefficients $V_n$ in the order of the multiplexed units $\hbox {MU}_n$ are presented below the structures. These structures are apparently different, and they do not occur in Figs. 5 or 6 either. We have also determined the number of identical and different structures for different values of the detection efficiency $V_D$ in the considered region of the transmission coefficients $V_r$ and $V_t$. We have found that the number of unique structures present for $V_D=0.8$ ($V_D=0.85$) but absent for $V_D=0.95$ is 18 (11), while there are 8 (5) unique structures present for $V_D=0.95$ and absent for $V_D=0.8$ ($V_D=0.85$).

**Fig. 8:**
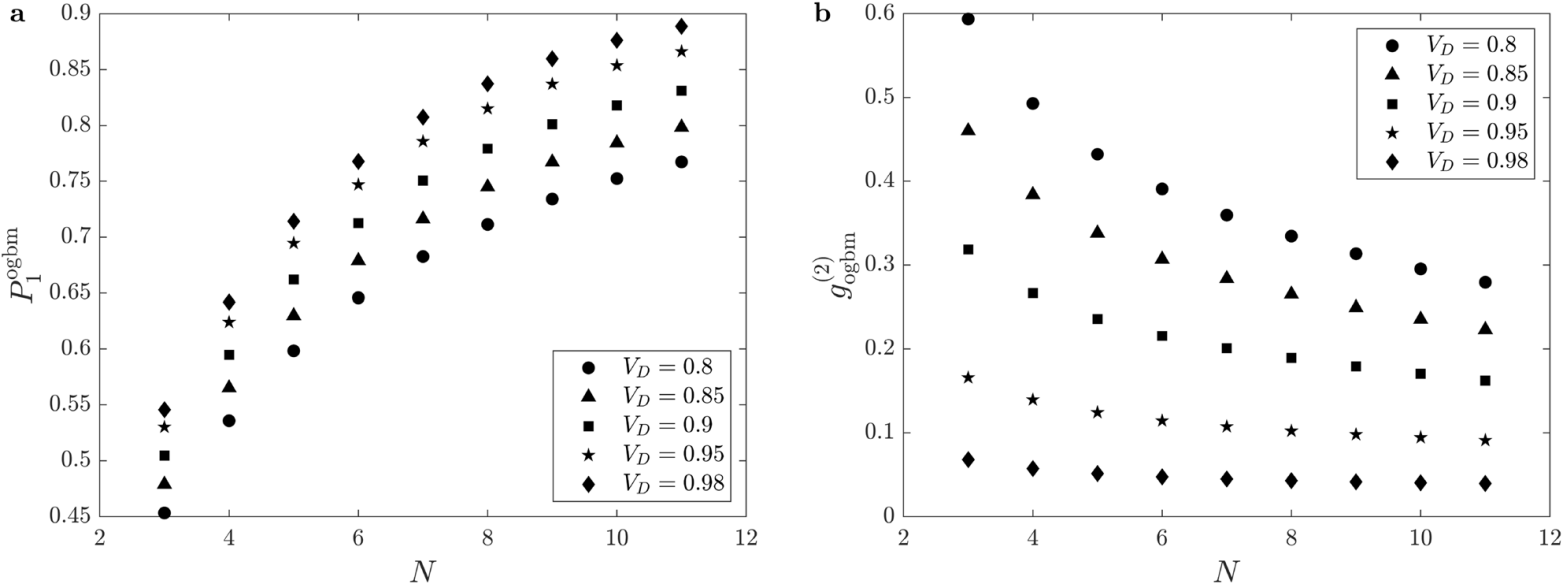
(**a**) The maximal single-photon probabilities $P_{1,\max }^{\textrm{ogbm}}$ and (**b**) the normalized second-order autocorrelation function $g^{(2)}_{\textrm{ogbm}}$ for SPSs based on OGBMs as functions of the number of multiplexed units *N* for the transmission coefficients $V_t=0.985$ and $V_r=0.99$, and for various values of the detector efficiency $V_D$.

Next, in Fig. 8 we show the maximal single-photon probabilities $P_{1,\max }^{\textrm{ogbm}}$ (Fig. 8a) and the normalized second-order autocorrelation function $g^{(2)}_{\textrm{ogbm}}$ (Fig. 8b) for SPSs based on OGBMs as functions of the number of multiplexed units *N* for the transmission coefficients $V_t=0.985$ and $V_r=0.99$, and for various values of the detector efficiency $V_D$. As it is expected, increasing the number of multiplexed units *N* leads to increasing values of the maximal single-photon probability $P_{1,\max }^{\textrm{ogbm}}$ and decreasing values of the second order autocorrelation function $g^{(2)}_{\textrm{ogbm}}$. Also, increasing the detector efficiency $V_D$ relevantly enhances the performance of the SPS. The maximal single-photon probability $P_{1,\max }$ that can be achieved for the detector efficiency $V_D=0.95$ and the number of multiplexed units $N=11$ is $P_{1,\max }=0.866$, while by modifying the detector efficiency to $V_D=0.98$ this probability can reach $P_{1,\max }=0.889$. These single-photon probabilities that can be achieved with experimentally realizable system sizes are quite promising compared to the probabilities that can be achieved with completely optimized SPSs based on previously considered multiplexers for the same loss parameters. For example, assuming the transmission coefficients $V_t=0.985$ and $V_r=0.99$, and the detector efficiency $V_D=0.95$, and using optimized system sizes in SPSs based on ASYM multiplexers, the maximal single-photon probability is $P_{1,\max }^{\textrm{asym}}=0.905$. However, the corresponding optimal number of the multiplexed units is considerably higher, $N_\text {opt}^{\textrm{asym}}=28$. The value of the second-order autocorrelation function $g^{(2)}$ that can be achieved for an SPS based on OGBM for the detector efficiency $V_D=0.95$ and the number of multiplexed units $N=11$ is $g^{(2)}=0.091$, while by modifying the detector efficiency to $V_D=0.98$ this value is $g^{(2)}=0.0395$. Note that these values can be also promising as they occur at the high single-photon probabilities mentioned above and using a multiplexer of small size.

Finally, we discuss the experimental realizability of SPSs based on OGBMs. Our method determines the optimal multiplexer structure built of PRs characterized by given losses for which the single-photon probability of the SPSs is the highest. The type of the PRs is not specified in our method; any of the realizations discussed in Section 2 can be used. The realization of SPSs based on OGBMs poses the same experimental challenges as those of SPSs based on any special type of spatial multiplexers^13,14,16,17,20,21^, no extra ones arise. A description of the various experimental realizations of spatially multiplexed SPSs can also be found in recent review papers^2,3^. A relevant problem regarding multiplexed SPSs is the realization of a system with optimal size, that is, increasing the number of MUs and the necessary PRs to the optimal values. This problem arises both in bulk and integrated optical realizations. Our results show that high single-photon probabilities can be achieved even for SPSs based on OGBMs with suboptimal system sizes. Nevertheless, integrated optical SPSs can be more advantageous in practical applications, due to their compactness and robustness.

## Conclusion

To improve the performance of spatially multiplexed single-photon sources, we have developed a method for optimizing the structure of general binary-tree multiplexers realized with asymmetric photon routers. Our procedure systematically considers all possible binary-tree multiplexers that can be constructed using a certain number of photon routers. Our optimization procedure selects the multiplexer structure that leads to the highest single-photon probability for a given set of loss parameters characterizing the system. We have determined the optimal general binary-tree multiplexers for experimentally realizable values of the transmission coefficients of the photon routers and that of the detector efficiency and for the number of multiplexed units $N=11$. As it is expected, single-photon sources based on optimal general binary-tree multiplexer yield higher single-photon probabilities compared to what can be achieved with single-photon sources based on any other multiplexer considered in the literature. Our approach improves the performance of multiplexed single-photon sources even for small system sizes which is the typical situation in current experiments.

## Data Availability

The datasets used and/or analysed during the current study are available from the corresponding author on reasonable request.
